# Do the Washington Panel recommendations hold for Europe: investigating the relation between quality of life versus work-status, absenteeism and presenteeism

**DOI:** 10.1186/1478-7547-12-24

**Published:** 2014-11-24

**Authors:** Saskia Knies, Annelies Boonen, Johan L Severens

**Affiliations:** Department of Health Services Research, School for Public Health and Primary Care (CAPHRI), Maastricht University, Maastricht, the Netherlands; National Health Care Institute, PO Box 320, 1110 AH Diemen, the Netherlands; Institute of Health Policy & Management, Erasmus University Rotterdam, Rotterdam, the Netherlands; Department of Internal Medicine, Division of Rheumatology, School for Public Health and Primary Care (CAPHRI), Maastricht University Medical Center+, Maastricht, the Netherlands; Institute of Medical Technology Assessment (iMTA), Erasmus University Rotterdam, Rotterdam, the Netherlands

**Keywords:** Quality of life, Productivity losses, Valuation, Relation

## Abstract

**Background:**

The question of how to value lost productivity in economic evaluations has been subject of debate in the past twenty years. According to the Washington panel, lost productivity influences health-related quality of life and should thus be considered a health effect instead of a cost to avoid double counting. Current empirical evidence on the inclusion of income loss when valuing health states is not decisive. We examined the relationship between three aspects of lost productivity (work-status, absenteeism and presenteeism) and patient or social valuation of health-related quality of life (HRQoL).

**Methods:**

Cross-sectional survey data were collected from a total of 830 respondents with a rheumatic disorder from four West-European countries. Health-related quality of life was expressed in either the European societal utility using EQ-5D-3L or the patient valuation using EQ-VAS. The impact of work-status (four categories), absenteeism (absent from paid work during the past three months), and presenteeism (QQ method) on EQ-5D utilities and VAS scores was examined in linear regression analyses taking into account demographic characteristics and disease severity (duration, pain and restriction).

**Results:**

The relationship between work-status, absenteeism or presenteeism and HRQoL was stronger for patient valuation than societal valuation. Compared to work-status and presenteeism the relationship between absenteeism and HRQoL was even less explicit. However, results for all measures of lost productivity are only marginally significant and negligible compared to the influence of disease-related restrictions.

**Conclusions:**

This survey study in patients with a rheumatic disorder in four European countries, does not fully support the Washington panel’s claim that lost productivity is a significantly related with HRQoL, and this is even more apparent for absenteeism than for work-status and presenteeism. For West-European countries, there is no reason, to include absenteeism in the QALY. Findings need to be confirmed in other disease areas.

## Background

A growing number of jurisdictions require economic evaluations for decisions about reimbursement of new technologies, particularly pharmaceuticals. For this reason, several jurisdictions have developed guidelines for pharmacoeconomic evaluations, outlining how to submit official reimbursement applications. However, these recommendations vary widely [1, 2]. One of the main aspects influencing the outcome of an economic evaluation is the recommended perspective, as this largely determines the costs and effects to be included [3, 4].

In relation to the discussion on the perspective, the way to include the patient’s lost productivity in economic evaluations is being debated [5]. When accepting the societal perspective, the next question is the approach in how to include lost productivity, as costs (nominator) or as effect (denominator) [4–6]? The main advocate of incorporating lost productivity of the patient in the effect side is the Washington Panel [7], whereas other researchers’ have made a plea for the cost side [4]. Thus, all in all there is no consensus on how to value lost productivity in costs. Still most national pharmacoeconomic guidelines containing recommendations on inclusion of lost productivity adhere to the cost side [8]. Nevertheless, the discussion on the valuation of lost productivity and the relation between health-related quality of life and productivity is still continuing. So far, however, research in this field is scarce and concentrates on the issue whether people take personal loss of income or lost production for society into account when valuing health states [9].

The Washington Panel distinguishes five components of lost productivity, two of which should be included in the QALY or denominator, namely I) the effects of lost productivity on the sick employee and II) the effects of lost leisure time on the sick employee. In contrast, III) the effect on the employer (recruitment and training of new employee) and IV) the external effects should be valued at the cost side or in the numerator. Lastly, V) the costs related to mortality on lost productivity should be presented separately and should not be included in the cost-effectiveness ratio as the denominator already contains the effects of mortality when the QALY is the measure of effectiveness [4, 7]. According to the Washington Panel lost productivity influences a patient’s health-related quality of life and thus can be considered a health effect, which implies that the full impact of morbidity should be included in the denominator [7]. As the denominator of the cost-effectiveness ratio captures the full value of health effects, inclusion of lost productivity in the numerator will result in double-counting of lost productivity [10]. Still, this will only be the case when the QALY is used as a measure of benefit [7]. It can be difficult for both the patient and the general public, however, to comprehend that the impact of the disease on work should be taken into account when valuing health states, and furthermore to distinguish between the impact of illness on lost productivity for society and the impact of the illness on the role function as a worker for the individual employee [7]. Moreover, costs might be underestimated by considering net wages rather than gross wages as a proxy for productivity [11, 12]. In addition, when valuing health, there is not always a link between reduced productivity and loss of income or at least not a proportional loss of income due to receiving social benefits to compensate for the sick employee’s reduction of salary [4, 11]. In relation to this, it is noteworthy that the profiles of health do not describe the impact of disease on work or income. This is especially a problem when someone from the general public is valuing a health state. However, the Washington Panel indicates that the preference weights to calculate the utilities from health states should be based on community or societal preferences instead of patient or provider preferences. It is argued that a representative sample of fully informed members of the community will, according to the Washington Panel, give the best representation of society’s preferences for particular health states [7, 10]. Last but not least, the Washington Panel does not explain clearly whether presenteeism as a type of lost productivity should be valued as a kind of costs or effect.

To our knowledge, so for it has not yet been investigated to what extent aspects of lost productivity such as being work disabled, being on sick leave and having presenteeism, influence the assessment of the quality of life of people with chronic health problems. The aim of our study is to examine the relationship between three aspects of lost productivity (work-status, absenteeism and presenteeism) and direct patient or indirect societal perspective of health-related quality of life (HRQoL). A population of persons with rheumatic disorders was chosen as the case sample, since in rheumatic disorders the influence of disease on productivity is high and therefore relationships are easier to study.

## Methods

### Population and questionnaire

Data were collected in March 2010 using an online questionnaire in four West-European countries, namely the Netherlands, the United Kingdom, France and Germany. The data were collected within the framework of another study, on between-country differences in the self-reported lost productivity of people with a rheumatic disorder. The data collection was carried out by the research organization TNS NIPO and all respondents were voluntary members of their patient panels of people with a rheumatic disorder. Eligible members of these panels received an e-mail message with a link to the online questionnaire. Persons were considered to be eligible if they themselves had stated to have a rheumatic disorder and were between twenty and seventy years of age. Although at the time of the study the retirement age in all countries was sixty-five or younger, people up to seventy years of age could participate if they were still working or wishing to have paid employment. A total of two hundred respondents per country with a rheumatic disorder were included. Overall, eight hundred-thirty respondents filled out the questionnaire as two hundred-thirty respondents in the Netherlands filled out the questionnaire. More details about the procedure and selection of respondents have been reported elsewhere [13].

This cross-sectional survey was carried out in competent subjects without any intervention. The study did not involve any form of randomization, testing medical treatments or obtaining humans material or invasion of the study participant’s integrity. According to the Dutch Medical Research Involving Human Subjects Acts (see http://www.ccmo.nl) approval by a medical-ethics committee is waived for such studies. This holds true for the three other countries as well, and therefore no approval of ethics committees in any of the four study countries was needed. Furthermore, participation was voluntary. The collected survey data and results were non-traceable to individual respondents.

The questionnaire used in the survey was composed of a number of validated self-report questionnaires. Health-related quality of life was measured using the EuroQol-5D-3L (EQ-5D) including the Visual Analogue Scale (VAS) [14]. Demographic (age, gender, work-status), work characteristics and lost productivity (both absenteeism and presenteeism) were measured using the Productivity and Disease Questionnaire (PRODISQ), which is a modular questionnaire developed to measure lost productivity in paid labour [15, 16]. Several questions were added to assess absenteeism, including having been absent in the last three months, currently absent and the number of days absent in the last three months. Presenteeism in PRODISQ is assessed using the QQ approach, by which respondents indicate how well (quality) and how much (quantity) work they carried out on their last working day on a ten point scale (10 indicating normal quality and quantity). An overall presenteeism score was created from both presenteeism scales [15, 16]. Questions about severity of the rheumatic disorder were selected from the Dutch-Arthritis Impact Measurement Scales-2 (Dutch-AIMS2), an instrument to assess the health status of people with a rheumatic disorder. As part of the AIMS, respondents were asked to indicate the level of experienced restrictions due to their rheumatic disorder in the last six months on a three point scale (not restricted, somewhat restricted and seriously restricted) and to rate pain due to the rheumatic disorder on a scale from 0 to 100 (no pain to severe pain). In addition, the respondents were asked to state when they received the diagnosis of their rheumatic disorder [17].

Respondents filled out the online questionnaire in the language of the specific country, for example respondents in Germany received the German-language questionnaire. Validated translations of questionnaires were utilized when available in the relevant language (EQ-5D-3L, AIMS). For questions and questionnaires for which no validated translation was available (demographics and PRODISQ), a translation from Dutch to language of interest by professional translators was provided [13].

### Statistical analyses

Prior to analysis, some variables were redefined or calculated. The utilities of the EQ-5D-3L health states were calculated using the European value set derived from an experiment respondents from the general population in six European countries [14], generally overlapping with the countries in our survey. Work-status of the respondents was classified categorised into four categories: I) having a paid job; II) having a paid job, but currently absent due to illness; III) no paid job, but receiving disability benefit; and IV) unemployed and not receiving disability benefit. All variables were checked for missing or abnormal values and for distribution by plotting residuals and inspecting if the data fulfilled the assumptions of normality. Missing data were found by the variables ‘currently absent’ and ‘number of days absent in the last three months’. All missing data could be imputed using the answer on the variable ‘having been absent in the last three months’, since no data were missing for the latter variable.

The demographic, disease and work characteristics of the sample are presented using descriptive statistics for the total group and sub-groups defined by work-status and were compared across groups using one-way ANOVA for the continuous independent variables and χ^2^- tests for the categorical variables. Linear regression analyses were performed in several models to examine the influence of I) work-status, II) absenteeism, III) presenteeism and IV) absenteeism and presenteeism together on either direct patients’ VAS-score or EQ-5D utility score, which reflects the societal preference. Possible explanatory variables were checked on normality, colinearity between covariates was explored as well as linearity between covariates and quality of life data (both VAS as well as EQ-5D data). All remaining variables were included as possible confounders or independent factors in the models.

## Results

### Characteristics of the respondents

Table 1 lists the characteristics of the whole sample (830 respondents) and of the different work-status categories. The largest group (57.6%) is the group with a paid job not on sick leave; 21.3% are unemployed without disability benefit; 13.7% were receiving disability benefit; and 7.3% reported being absent.

**Table 1:** **Characteristics of the respondents (n = 830)**

		Total population	Working, not reporting abenteeism ^1^	Working, currently absenteeism ^2^	Not working, getting disability benefit ^3^	Not working, unemployed ^4^
Number (%)		830	478 (57.6%)	61 (7.3%)	114 (13.7%)	177 (21.3%)
Male (%)		295 (35.5%)	182 (38.1%)	24 (39.3%)	38 (33.3%)	51 (28.8%)
Age (range)		49 (20-70)	47 (20-65)^3,4***^	46 (23-63)^3**, 4***^	51(25-64)^1***, 2**^	52 (24-70)^1,2***^
EQ-5D (European) (range -0.074-1)		0.743 (0.05-1.00)	0.794 (0.21-1.00)^2,3,4***^	0.685 (0.05-1.00)^1***,3*^	0.600 (0.05-1.00)^1,4***,2*^	0.717 (0.05-1.00)^1,3***^
VAS EQ-5D (range 0 -100)		63.64 (0-100)	69.16 (0-100)^2,3,4***^	56.34 (2-100)^1***^	49.93 (7-95)^1,4***^	60.05 (0-98)^1,4***^
Pain (range 0 -100)		43.30 (0-100)	39.07 (0-100)^2,4*,3***^	48.57 (0-95)^1*^	55.18 (2-100)^1***,4*^	45.23 (0-100)^1,3*^
Disease duration (range)		12.61 (0-56)	11.73 (0-55)^3**,4*^	10.67 (1-36)^3*^	15.25 (1-52)^1**,2*^	13.95 (1-56)^1*^
Seriously restricted (%)		155 (18.7%)	44 (9.2%)^2,3***,4**^	25 (41%)^1,4***^	51 (44.7%)^1,4***^	35 (19.8%)^1**,2,3***^
Somewhat restricted (%)		530 (63.9%)	320 (66.9%)^3*^	34 (55.7%)	60 (52.6%)^1*^	116 (65.5%)
Country of residence						
	the Netherlands (%)	230 (27.7%)	156 (32.6%)	17 (27.9%)	32 (28.1%)	25 (14.1%)
	United Kingdom (%)	200 (24.1%)	105 (22%)	111 (18%)	36 (31.6%)	48 (27.1%)
	France (%)	200 (24.1%)	114 (23.8%)	15 (24.6%)	23 (20.2%)	48 (27.1%)
	Germany (%)	200 (24.1%)	103 (21.5%)	18 (29.5%)	23 (20.2%)	56 (31.6%)
Not working due to RD (%)		160 (9.3%)	NA	NA	103 (90.4%)	57 (32.2%)
Having been absent in the last 3 months (%)		167 (20.1%)	106 (22.2%)^2***^	61 (100%)^1***^	NA	NA
Number of days absent (range 0-92)		18.89 (0-92)	8.58 (0-92)^2***^	36.80 (1-91)^1***^	NA	NA
Presenteeism – quantity (range 0-10)		8.60 (0-10)	8.81 (0-10)^2***^	7.02 (0-10)^1***^	NA	NA
Presenteeism – quality (range 0-10)		8.83 (0-10)	8.94 (0-10)^2***^	8.03 (0-10)^1***^	NA	NA
Overall presenteeism – quantity * quality (range 0-100)		78.80 (0-100)	80.91 (0-100)^2***^	62.30 (0-100)^1***^	NA	NA

^1^all respondents with a paid job who are when filling out questionnaire not absent.

^2^all respondents with a paid job who are when filling out questionnaire absent.

^3^all respondents without a paid job who receive disability benefit.

^4^all respondents without a paid job who do not receive disability benefit.

NA: not applicable.

RD: rheumatic disorder.

*p <0.10; **p <0.01; ***p <0.001.

Respondents who had a paid job but were currently absent from work tended to be more frequently males, younger, living in the Netherlands and to have a shorter disease duration. Those who were not absent had less pain, were less often seriously restricted and had better quality of life based on both VAS and EQ-5D. In addition, they reported a lower average number of days absent in the last three months than the respondents who were absent, and higher presenteeism scores on their last working day, which implies that they were close to fully productive. However, because respondents answered these questions on their last working day, there is a risk of recall bias for respondents who are absent for a longer period of time. Respondents who receive disability benefit had the lowest quality of life, both measured with the VAS and EQ-5D. Furthermore, they had more pain, were more frequently seriously restricted, more often living in the Netherlands or in the United Kingdom and had a longer disease duration. The unemployed respondents were clearly the oldest, more often females and living in Germany. However, they were less often seriously restricted than respondents on sick leave, but they had longer disease duration. About one third of them indicated they did not work due to their rheumatic disorder.

### Relation between quality of life and work-status

Table 2 presents the results of the linear regression analyses that explored the influence of work-status on both the VAS scores (self-reported health) and the EQ-5D derived utilities. The model for the VAS scores has an R^2^ of 0.308, which implies that 30.8% of the variance of the VAS scores are explained by the model. The R^2^ of the model for the EQ-5D utilities was 0.354 (explaining 35.4%).

**Table 2:** **Linear regression exploring the impact of work-status on VAS scores and EQ-5D utilities (n = 830)**

		VAS scores (self-reported health)			Utilities European EQ-5D value set		
		B	Std. error	Beta	B	Std. error	Beta
Work status							
	Absent versus working	-3.882	2.524	-.047	-.027	.021	-.037
	Disabled versus working	-9.504	2.021	-.152***	-.101	.017	-.186***
	Unemployed versus working	-6.118	1.640	-.117***	-.050	.014	-.109***
Age (years)		.104	.065	.049	.001	.001	.062*
Gender (1: male, 2: female)		1.501	1.326	.033	.012	.011	.031
Pain (range 0-100)		-.026	.024	-.034	-.001	.000	-.168***
Disease duration (years)		-.111	.064	-.052*	-.001	.001	-.033
Restriction due to disease							
	Seriously restricted versus not restricted	-32.649	2.346	-.592***	-.247	.020	-.514***
	Somewhat restricted versus not restricted	-15.443	1.780	-.345***	-.071	.015	-.184***
		Note: R-square .308			Note: R-square .354		

*p <0.10; **p <0.01; ***p <0.001.

The variables age, pain due to rheumatic disorder, years of rheumatic disorder, restriction due to disease and work-status were all negatively correlated to quality of life. Including the same covariates, respondents with sickness absence scored (Beta) -0.047 worse on VAS and -0.037 on EQ-5D compared to respondents with a paid job after correcting for other variables. Respondents who receive disability benefit are expected to score -0.152 worse on VAS and -0.186 points on EQ-5D compared to the currently working respondents. Unemployed respondents scored -0.117 worse on VAS and -0.109 on EQ-5D compared to the working respondents. Seriously restricted respondents had the lowest quality of life both on the VAS and EQ-5D. For both models it was checked whether the inclusion of work-status had a significant influence. For the VAS model the F-change was (3, 820) =9.47, *p* <0.0001. The F-change for the EQ-5D European model was (3, 820) =3.11, *p* <0.0001.

### Quality of life and absenteeism

Table 3 shows the results of the linear regression on the relation between the quality of life and absenteeism for all respondents with paid work (n =537). The influence of absenteeism (currently absent or absent in the last three months but not now versus not absent in the last three months) was explored while correcting for other possible influential variables. The R^2^ of the model explaining the VAS score was 0.263 and the R^2^ of the model explaining EQ-5D utilities was 0.310. While age and duration of the rheumatic disorder had no statistically significant influence on quality of life, pain and restrictions were major determinants for both patient reported VAS health and indirect societal value for health. Not having been absent in the last three months resulted in a significantly higher quality of life compared to being currently absent with a higher VAS score of 0.068 point.

**Table 3:** **Linear regression on influence of absenteeism restricted to respondents with paid employment (n = 537)**

		VAS score (self-reported health)			Utilities European EQ-5D value set		
		B	Std. error	Beta	B	Std. error	Beta
Absenteeism							
	Currently absent versus not absent at all	-4.365	2.595	-.068*	-.032	.020	-.061
	Absent in last three months versus not absent at all	-2.123	1.975	-.041	-.017	.015	-.041
Age (years)		.038	.081	.018	.000	.001	.010
Gender (1: male, 2: female)		.242	1.581	.006	.021	.012	.061*
Pain (range 0-100)		-.081	.029	-.112**	-.001	.000	-.223***
Disease duration (years)		-.126	.086	-.056	-.001	.001	-.034
Restriction due to disease							
	Seriously restricted versus not restricted	-30.595	2.978	-.502***	-.238	.023	-.485***
	Somewhat restricted versus not restricted	-13.323	2.013	-.311***	-.064	.016	-.185***
		Note: R-square .263			Note: R-square .310		

*p <0.10; **p <0.01; ***p <0.001.

### Quality of life and presenteeism

Table 4 shows the results of the linear regression in which the correlation between presenteeism (product of quality and quantity) and quality of life was explored for persons with paid work, including those currently absent, correcting for possible influential variables. The R^2^ of the model explaining the VAS scores was 0.290 and the R^2^ of the model explaining EQ-5D utilities was 0.322. While age and gender had no independent influence on quality of life, disease duration contributed to the variation in patient reported VAS health, and pain and restrictions were major determinants for both patient reported VAS health and indirect societal value for health. Each point of improvement on overall presenteeism increased VAS by 0.183 point and EQ-5D by 0.128 point.

**Table 4:** **Linear regression on influence of presenteeism restricted to respondents with paid employment (n = 537)**

		VAS score (self-reported health)			Utilities European EQ-5D value set		
		B	Std. error	Beta	B	Std. error	Beta
Overall presenteeism (range 0-100)		.137	.028	.183***	.001	.000	.128**
Age (years)		.026	.079	.012	.000	.001	.008
Gender (1: male, 2: female)		-.083	1.552	-.002	.019	.012	.056
Pain (range 0-100)		-.075	.029	-.103**	-.001	.000	-.218***
Disease duration (years)		-.160	.085	-.071*	-.001	.001	-.045
Restriction due to disease							
	Seriously restricted versus not restricted	-29.971	2.811	-.492***	-.237	.022	-.484***
	Somewhat restricted versus not restricted	-13.065	1.962	-.305***	-.063	.015	-.183***
		Note: R-square .290			Note: R-square .322		

*p <0.10; **p <0.01; ***p <0.001.

### Quality of life and absenteeism and presenteeism

Table 5 shows the results of the linear regression exploring the influence of absenteeism and overall presenteeism (product of quality and quantity) on quality of life in persons with paid work (including those currently absent), correcting for possible influential variables. The R^2^ of the model explaining the VAS scores was 0.291 and the R^2^ of the model explaining EQ-5D utilities was 0.324.

**Table 5:** **Linear regression on influence of absenteeism and presenteeism restricted to respondents with paid employment (n = 537)**

		VAS score (self-reported health)			Utilities European EQ-5D value set		
		B	Std. error	Beta	B	Std. error	Beta
Absenteeism							
	Currently absent versus not absent at all	-2.321	2.585	-.036	-.020	.020	-.040
	Absent in last three months versus not absent at all	-1.529	1.942	-.030	-.014	.015	-.033
Overall presenteeism (range 0-100)		.132	.029	.176***	.001	.000	.120***
Age (years)		.017	.079	.008	5.02 *10^-5^	.001	.003
Gender (1: male, 2: female)		-.084	1.554	-.002	.019	.012	.056
Pain (range 0-100)		-.075	.029	-.103**	-.001	.000	-.217***
Disease duration (years)		-.159	.085	-.070*	-.001	.001	-.044
Restriction due to disease							
	Seriously restricted versus not restricted	-29.124	2.939	-.478***	-.230	.023	-.468***
	Somewhat restricted versus not restricted	-12.806	1.978	-.299***	-.061	.016	-.176***
		Note: R-square .291			Note: R-square .324		

*p <0.10; **p <0.01; ***p <0.001.

While age, gender and absenteeism had no independent influence on quality of life, disease duration contributed to the variation in patient reported VAS health. However, pain due to the rheumatic disorder and restrictions were major determinants for both patient reported VAS health and indirect societal value for health. Each point of improvement on overall presenteeism increased VAS by 0.176 point and EQ-5D by 0.120 point.

## Discussion

The question whether the approach recommended by the Washington Panel is justified cannot be answered with a simple yes or no. The results of this study show that the situation is more complex. First, although the relations between work-status and presenteeism on the one hand and health-related quality of life on the other hand (both patient and societal valuation) are significant, the relationship between absenteeism and health-related quality of life is less clear. Secondly, while there is a significant relationship between patient reported health-related quality of life and work-status, the relation between the societal valuation and absenteeism is not significant. Furthermore, when presenteeism and absenteeism are both included in the analyses, the influence of absenteeism is no longer significant. Since the Washington Panel recommends using societal preferences when calculating the QALY, the monetary valuation of absenteeism will probably not result in double counting as the Washington Panel advocates. Disease related restrictions seem to affect quality of life the most, especially for seriously restricted respondents. In addition, also pain due to the rheumatic disorder is significantly correlated with quality of life.

### Comparison with the literature

In previous research the relation between health-related quality of life and lost productivity was investigated by either looking whether respondents take into account the income effects of lost productivity when valuing health states, or whether respondents value health states differently when they are explicitly asked to take into account lost productivity or income effects due to lost productivity. Our results are generally in line with the conclusions from these previous studies. Based on a review by Tilling et al. [18] concluded that the empirical evidence on this issue is not decisive as the existing studies show a number of inconsistencies on whether or not the general population and patients spontaneously include income effects when valuing health states. Shiroiwa et al. [19] investigated the influence of income reduction on utility scores by asking respondents from the Japanese general population to value EQ-5D health states whereby respondents were given different instructions on whether or not to consider income reduction [19]. The maximum difference between the different types of instructions in the valuation of the health states was estimated to be 0.05. Therefore the authors concluded that the impact of double counting is negligible, seeing that the effect of income on utility scores does not only reflect wage loss [19]. Krol et al. [20] tried to predict patient’s productivity from health states by asking a sample of the Dutch general population to estimate the expected level of productivity for several EQ-5D health states. A linear relationship between utility score and productivity was not found as different levels of the EQ-5D had a different impact on quality of life than on productivity. Especially lost productivity due to presenteeism was overestimated in their prediction model. Thus, previous research shows that the relationship between quality of life, income loss and lost productivity is not clear-cut, which is in accordance with our results.

As far as we know, our study was the first to look simultaneously at the relationship between three different components of lost productivity: work-status, absenteeism and presenteeism, and the combination of absenteeism and presenteeism. Earlier studies did not take into account disease-specific characteristics that could influence the health-related quality of life, such as disease duration, pain and restriction due to disease, when investigating the relationship between quality of life and lost productivity. By including these covariates into the analyses the influence of lost productivity is not biased by disease specific characteristics.

If the findings of our study should prove to be generalizable to other disease areas or to the general population, then the recommendations in the national pharmacoeconomic guidelines regarding the valuation of lost productivity need not be changed. As mentioned before most national pharmacoeconomic guidelines recommend valuing lost productivity as costs. This is especially true for recommendations about the valuation of absenteeism [8]. In the present study the relationship between absenteeism and the societal valuation of health-related quality of life was not statistically significant and the relationship with the patient valuation had a small significant effect.

### Limitations of the study

Several limitations of this study should be addressed related to both the study sample and the explorative nature of the study. First, we included only respondents with a self-reported rheumatic disorder to create a sample with a relatively homogeneous disease state in lost productivity is often encountered. Findings for other diseases and patient populations could well be different; therefore the generalizability to other disease areas or to the general population might be limited. Secondly, distribution of the eight hundred-thirty respondents over the four work-status groups was unequal, with the largest number of respondents in the group of having a paid job, not reporting absenteeism. Still, this total number of respondents was large enough to statistically significant differences. However, the numbers of respondents per group were too low to perform analyses for each country separately. Third, due to the limited number of explanatory variables in the survey it was not possible to explore the role of other factors that might affect the relation between the different types of lost productivity and quality of life [19], such as job and workplace related factors, but also additional disease characteristics or personal factors such as coping or self-management styles [13]. This is especially true for presenteeism. Still about 30% of the health-related quality of life could be explained by the statistical models. This study indicates that additional research is needed to better understand the relationship between presenteeism and quality of life. Fourth, in our study we only had cross-sectional data and therefore it was not possible to explore possible relations between quality of life and lost productivity over time.

## Conclusion

The recommendations of the Washington Panel to include lost productivity due to sick leave in the denominator have stimulated research projects investigating the relationship between quality of life and absenteeism. However, limited research has been carried out on the influence of presenteeism on quality of life, seeing that presenteeism can result in lost productivity. Therefore, additional research is needed to better understand the relationship between presenteeism and quality of life. This study showed that especially being absent has marginal influence on quality of life. As a consequence, results provide further support for including absenteeism in the costs side in economic evaluations as this will not lead to double-counting of lost productivity. As work-status and presenteeism seem to have at least some influence on quality of life, the extent towards these aspects should or should not be included in the costs remains more open. So for the time being, it can be concluded that the Washington Panel recommendations on lost productivity do not seem to hold for Europe.

## Authors’ information

The views expressed in this article are those of the authors and should not be attributed to the authors’ employers.
